# Oral candidiasis in patients with renal transplants

**DOI:** 10.4317/medoral.18658

**Published:** 2013-02-05

**Authors:** Rosa M. López-Pintor, Gonzalo Hernández, Lorenzo de Arriba, Amado de Andrés

**Affiliations:** 1 DDS, PhD. Assistant Professor. Department of Oral Medicine and Orofacial Surgery, Faculty of Odontology, Complutense University, Madrid, Spain. Plaza Ramón y Cajal s/n, 28040 Madrid, Spain; 2 MD, DDS, PhD. Professor. Department of Oral Medicine and Orofacial Surgery, Faculty of Odontology, Complutense University, Madrid, Spain. Plaza Ramón y Cajal s/n, 28040 Madrid, Spain; 3 MD, DDS, PhD. Assistant Professor. Department of Oral Medicine and Orofacial Surgery, Faculty of Odontology, Complutense University, Madrid, Spain. Plaza Ramón y Cajal s/n, 28040 Madrid, Spain; 4 MD, PhD. Assistant Professor. Department of Nephrology and Transplant Coordination, Hospital Universitario 12 de Octubre, Complutense University, Madrid, Spain. Avenida de Córdoba s/n, 28026 Madrid, Spain

## Abstract

Objectives: Oral candidiasis (OC) is a frequent oral lesion in renal transplant patients (RTPs). Despite the increased prevalence of OC in RTPs, no study has examined related risk factors. The aims of this study were to analyze the prevalence of and risk factors for OC in RTPs compared with age- and gender-matched healthy control group (HC) as well as determine the incidence of OC after transplantation. Study Design: We analyzed the prevalence and risk factors of OC in a group of 500 RTPs (307 men, 193 women, mean age 53.63 years) and 501 HC subjects (314 men, 187 women, mean age 52.25 years). Demographic and pharmacological data were recorded for all subjects. Incident cases of OC were ascertained retrospectively from outpatient clinical records only in the RTP group. Results: The prevalence of OC was 7.4% in RTPs compared with 4.19% in HC (P<0.03). The most frequent type of OC in the two groups was denture stomatitis. Statistical association was found between OC and age, mycophenolate mofetil dose and blood levels, dentures and tobacco. The multiple logistic regression model only chose for denture variable. According to the outpatient clinical records, 24 RTPs suffered OC during the first moth post-transplant. Severe lesions affecting the oral cavity and pharynx appeared in 79% of the OC cases. Conclusions: This study shows a lower prevalence of OC in RTPs than previous reports. Denture stomatitis was the most frequent OC prevalence form described in RTPs. Severe candidiasis is more frequent in the immediate posttransplant period. The presence of denture is an important risk factor of OC. These results emphasise the importance of adequate pre- and post-transplant oral health and denture cleaning and adjustment is recommended for these subjects to prevent this infection.

** Key words:**Oral candidiasis, immunosuppressive therapy, renal transplantation.

## Introduction

Solid-organ transplantation is a globally accepted procedure for patients with irreversible organ failure. This therapy is associated with different side-effects and the necessary immunosuppression leads to increased rates of infection, malignancy, and other complications (1-3).

Candidal infections are particularly prevalent after organ transplantation (4,5). Candida species can trigger infections of the bloodstream and esophagus as well as other organs in RTPs (6,7). Oral candidiasis (OC) can predispose such patients to esophageal candidiasis, an invasive form of infection with significant morbidity (5,8).

Previous studies have shown that RTPs have considerably higher prevalence of OC than healthy controls (HCs), and this condition is the most frequent oral infection in RTPs, with a prevalence ranging between 7.7% and 46.7% (8-13).

The transition of Candida from commensal to pathogen is often associated with predisposing factors. The systemic factors promoting OC in RTPs are immunosuppressant dose, diabetes mellitus, retransplantation, prolonged antibiotic use, leukopenia, xerostomic drugs, previous cytomegalovirus and/or human herpes virus 6 infections, and old age (2,6,14-18). Local factors either alter the mucosal barrier or diminish the quality or quantity of saliva to promote OC; they include poor oral hygiene, poor oral and dental condition, presence of dirty or poorly fitting dentures, antibiotic and/or local corticosteroid treatment, smoking, and physical and/or chemical trauma (18).

Recently, we observed that OC is the most frequent oral lesion in both RTPs and HCs (19). However, studies of the potential risk factors and predictors for this condition in RTPs are lacking. In addition, some studies suggested that oral infections, including candidiasis, are more severe in the immediate posttransplantation period (4,5), but cross-sectional studies of OC in RTPs did not support this finding (8-13). In this sense, a longitudinal design would be more reliable to analyze whether OC is more common in the immediate posttransplantation period and study its associated factors. Therefore, the aims of this study were to analyze the prevalence of and risk factors for OC in RTPs compared with age- and gender-matched HCs as well as determine the incidence of OC after transplantation.

## Material and Methods

-Study Population

Five hundred patients who underwent kidney transplantation between February 1989 and March 2007 were recruited from the outpatient Renal Transplant Clinic of Hospital 12 de Octubre in Madrid (307 men, 193 women; mean age = 53.63 ± 13.42 years, age range = 19–95 years; mean posttransplantation period = 59.66 ± 55.81 months, posttransplantation range = 1–330 months). HCs were recruited from the Julio Morate Health Center in Madrid (314 men, 187 women; mean age = 52.25 ± 15, age range = 20–93 years). The study was approved by the ethics committee of Hospital 12 de Octubre and written informed consent was obtained from all subjects.

All the HCs routinely received medical treatment for conditions such as hypertension, diabetes, or weight control, but none sought treatment for any oral mucosal disorder. HCs were excluded if they received a transplant, underwent immunosuppressant and/or corticosteroid therapy, or had renal diseases. Neither the RTPs nor the HCs received antibiotic, antifungal, or antiviral therapy one month before the assessment.

-Clinical Assessment

The oral mucosa and lips of all subjects were examined clinically by one investigator (R.M.L.P). The clinical diagnosis of candidiasis followed the criteria by Holmstrup and Axell (20), and the definitive diagnosis was supported by a positive response to antifungal treatment, positive candidal culture, and presence of candidal hyphae in stained smears. Incident cases of OC only in the RTPs were ascertained retrospectively from outpatient clinical records. The clinical diagnosis of OC in these cases was based on the clinical impression and supported by a positive response to therapy.

-Analyzed Variables

The data of both study populations were reviewed with regard to gender, age, diabetic history, antidepressant treatment, smoking habits, alcohol consumption, and presence of dentures. The time since transplantation, immunosuppressive treatment, and immunosuppressant dose were reviewed only for the RTPs. Further, hematologic studies were performed only for the RTPs, including testing for immunosuppressant blood levels, neutrophil and eosinophil counts, and hemoglobin and creatinine levels. These studies were conducted on the same day as the oral examination.

The subjects were asked about their smoking habits and current alcohol consumption. A smoking habit was measured in terms of cigarettes smoked per day. One cigar was assumed to be equal to four cigarettes. The intake of alcoholic beverages was expressed in units of alcohol per day (one unit = approximately 10 gm alcohol, half pint of beer, one small glass of wine, or one measure of spirits/hard liquor).

-Statistical Analysis

Statistical analysis was performed by using SPSS version 19.0 for Windows (IBM-SPSS, Inc., Chicago, IL). Differences between continuous variables and categorical variables were assessed by Student’s t-test and chi-square test, respectively. Multiple logistic regression analysis was conducted to explore the associations between OC and selected clinical variables. Variables were included in the model if they were predictors of the outcome (p < 0.05). The final model included group (RTPs/HCs), age, mycophenolate mofetil (MMF) dose and blood level, smoking habit, and presence of dentures. P < 0.05 was considered significant.

## Results

The clinical data of the study populations are presented in  Table 1. Overall, 4.8% of the men and 7.4% of the women had OC (P < 0.12). The subjects with OC were significantly older than those without OC (60.24 ± 12.44 vs. 52.49 ± 14.23, P < 0.0001). The presence of OC was statistically associated with smoking (P < 0.05) and the presence of dentures (P < 0.0001). OC appeared in 85.2% of the RTPs and 61.1% of the HCs who wore acrylic dentures as well as in 14.8% of the RTPs and 22.2% of the HCs who wore metallic dentures.

**Table 1:**
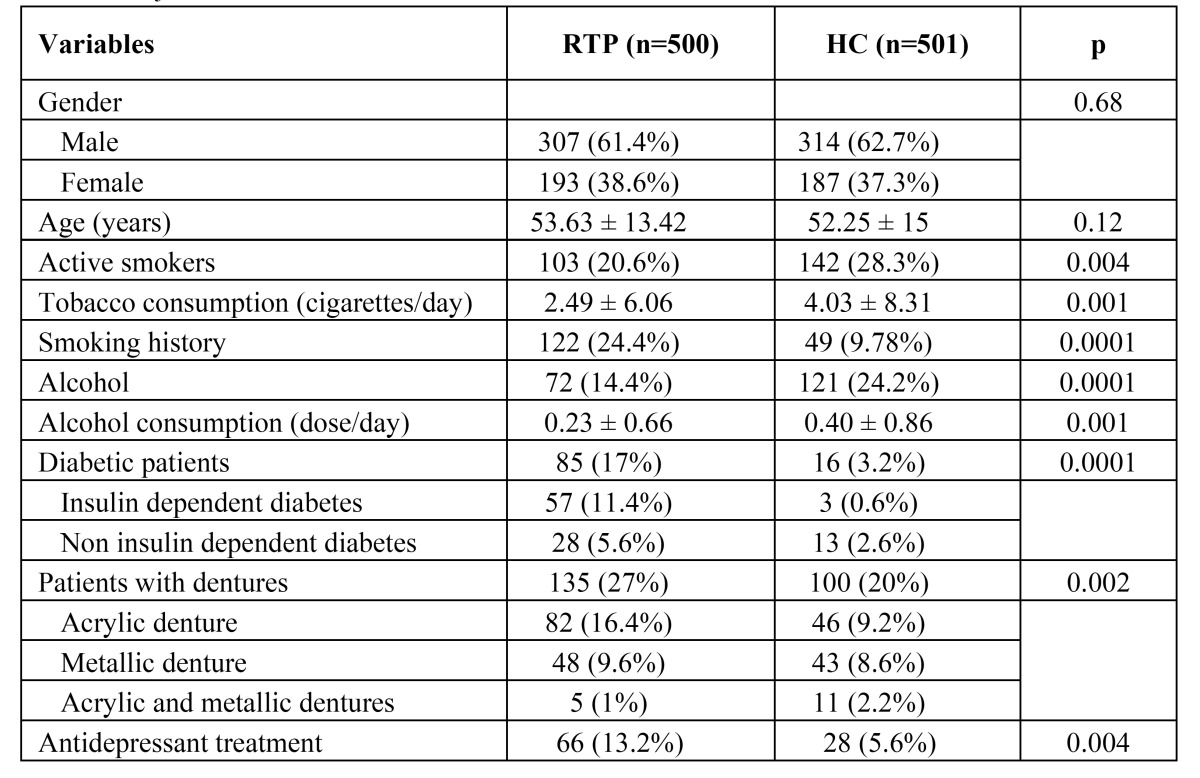
Subject variables and risk factors and the differences between RTPs and HC.

The different immunosuppressant schedules for the RTPs are listed in  Table 2. We did not find a statistical association between the presence of OC and the different immunosuppressant regimens (P < 0.83). The main regimens were prednisolone, tacrolimus, and MMF (35.13%); prednisolone, cyclosporine A (CsA), and MMF (16.22%); prednisolone and CsA (10.81%); and CsA alone (8.10%).

**Table 2:**
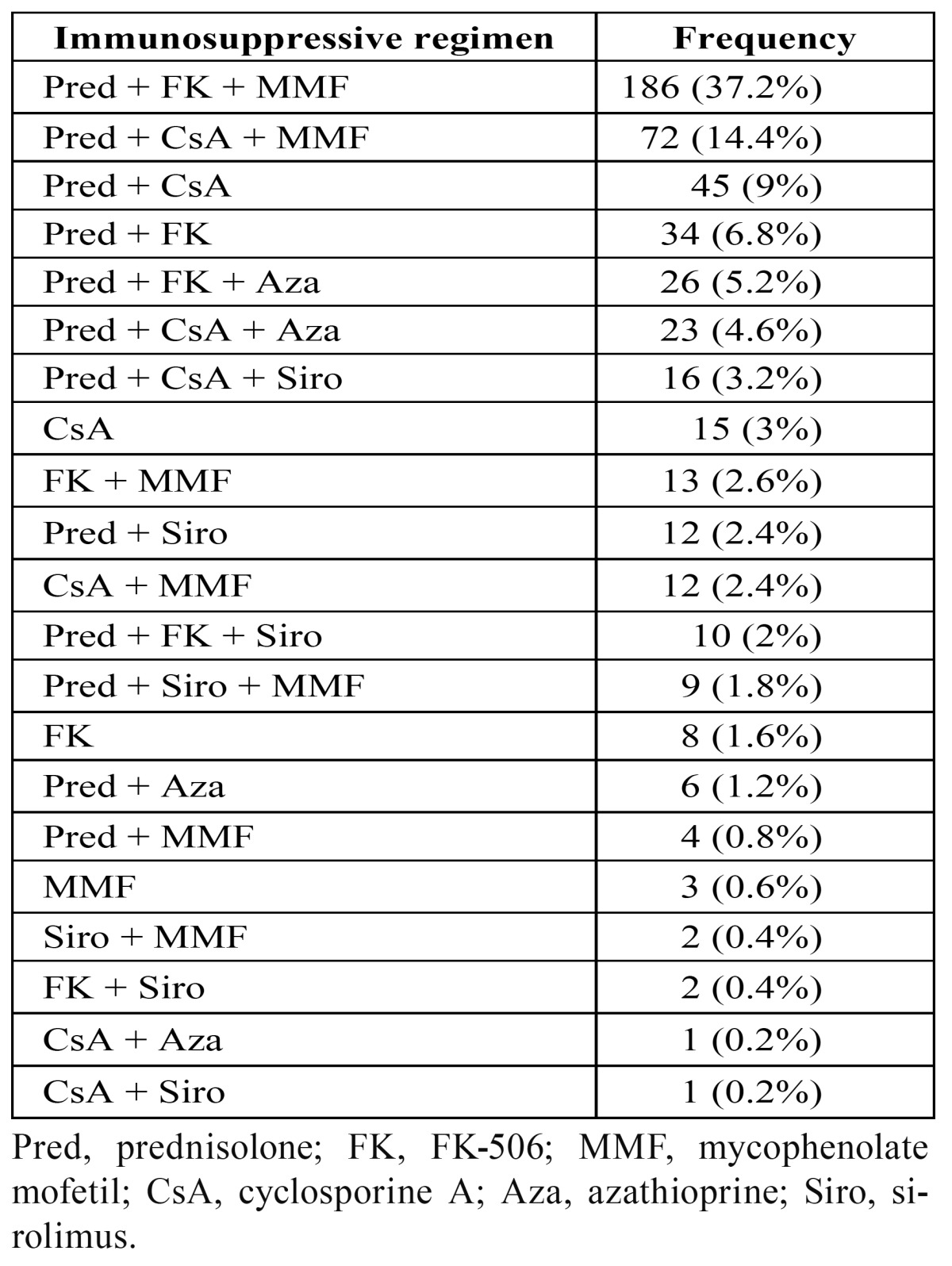
Immunosuppressive regimen of RTPs of study.

 Table 3 shows the mean immunosuppressant doses and blood levels as well as the number of RTPs administered these immunosuppressants. We found a statistical association between the presence of OC and the dose and blood level of MMF (P < 0.017 and P < 0.001, respectively). The RTPs with OC had received a mean MMF dose of 1177.08 ± 558.92 mg and had a mean MMF blood level of 4.37 ± 3.65 ng/ml. The mean MMF dose and blood level in the RTPs without OC were 960 ± 411.45 mg and 2.18 ± 1.71 ng/ml, respectively.

**Table 3:**
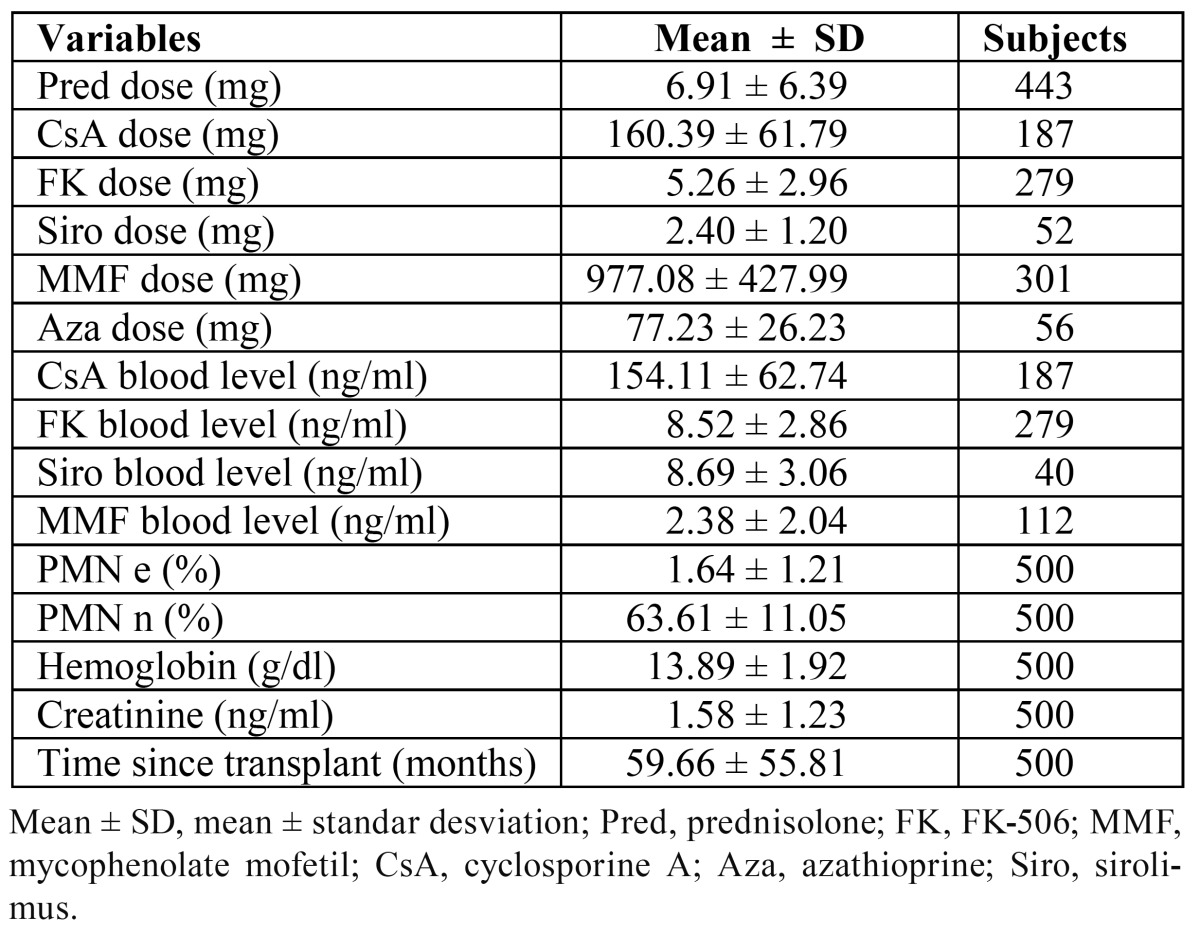
Mean ± standard deviation of inmunosuppresive drugs doses taken by the RTPs and laboratory test.

We found no statistical association between the presence of OC and the time since transplantation, dose and blood levels of the other immunosuppressants (i.e., apart from MMF), and the pharmacologic and hematologic variables in the RTPs. We did not find a statistical association between the presence of OC and diabetic history, antidepressant treatment, and alcohol consumption, in the RTPs and HCs.

The RTPs had significantly higher prevalence of OC than the HCs (7.4% vs. 4.19%, P < 0.03). Denture stomatitis was the most frequent form of OC in RTPs and HCs (5.4% and 3.6%, respectively), followed by angular cheilitis (1.6% and 0.2%) and pseudomembranous candidiasis (0.4% and 0.4%).

The odds ratio (OR) and 95% confidence interval (CI) in the final multiple logistic regression model chose only the presence of dentures as a significant variable. The RTPs and HCs who wore dentures were more prone to OC. Further, OC was more frequent in the RTPs who wore acrylic dentures (OR, 28.45; 95% CI, 13.64–59.34;P = 0.000) than in those who wore acrylic and metallic dentures (OR, 17.45; 95% CI, 4.29–70.88; P = 0.000) or metallic dentures alone (OR, 9.33; 95% CI, 3.77–23.09; P = 0.000).

 Table 4 provides information regarding the incident cases of OC in the RTPs. According to the outpatient clinical records, 24 RTPs (4.8%) suffered from OC in the first posttransplantation month, and 79.2% of these OC cases involved severe candidal lesions affecting the oral cavity and anterior pharyngeal pillars.

**Table 4:**
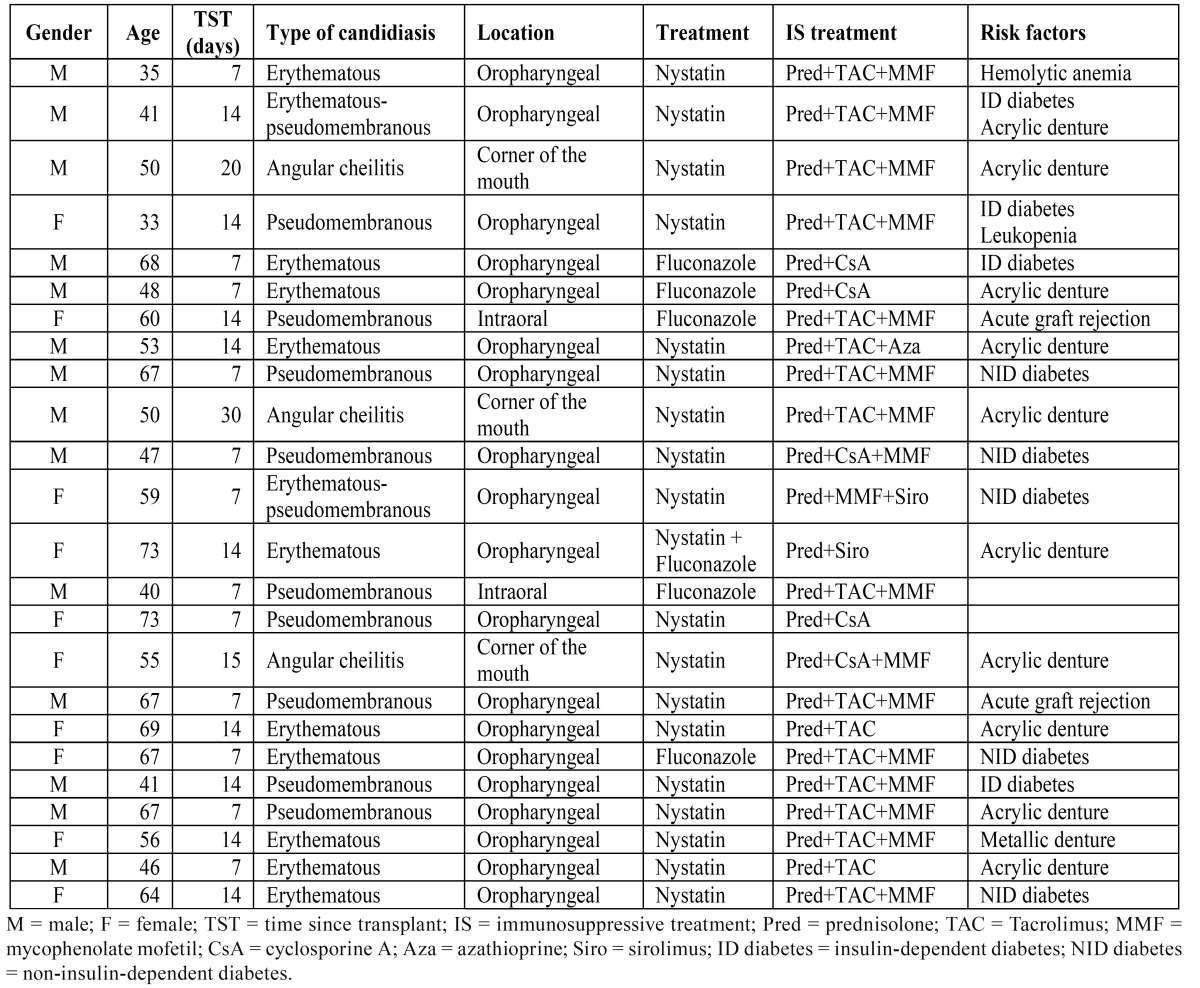
Incident cases of OC in RTPs.

## Discussion

In this study, we found higher prevalence of OC in the RTPs than in the HCs (7.4% vs. 4.19%). This result conforms to that reported by Dongari-Bagtzoglou et al. (8), a 7.7% OC prevalence in RTPs, suggests that the prevalence of OC in RTPs may be much lower than that previously reported (9.4–46.7%) (9-13). The difference may be attributable to variations in population characteristics, including the time after transplantation, immunosuppressant regimens, sample size, demographic causes, and overall health status of the populations examined. Importantly, immunosuppressive treatments have changed in the recent years. There have been dramatic shifts in baseline immunosuppression with increased use of induction agents and the nearly universal replacement of azathioprine by MMF. Further, tacrolimus use has increased whereas CsA use has fallen (21). Mammalian-target-of-rapamycin inhibitors are usually used nowadays as well (22). These changes in immunosuppressant protocols may be responsible for the discrepancies observed among different studies.

It is important to point out that OC is frequently asymptomatic (18). No patient with OC in our study had symptoms during diagnosis, justifying the importance of regular oral examinations, because OC can predispose in RTPs to esophageal candidiasis (5,8).

Infections in RTPs use to follow a predictable pattern in the posttransplantation period. This period is divided into three phases: the first posttransplantation month, 1–6 months posttransplantation, and 6 months posttransplantation and beyond. During the first posttransplantation month, the types of infection are similar to those seen postoperatively in immunocompetent patients. The causative organisms include Candida species, herpes simplex virus, and nosocomial bacteria. Thereafter, up to 6 months posttransplantation, recipients of solid-organ transplants have the maximum dysfunction of cellular immunity. Infections in this period are usually produced by intracellular or opportunistic pathogens, although OC and recurrent intraoral herpetic infections are also frequently seen. In the third phase after transplantation, the risk of infection varies and depends on the clinical course during the previous two phases and the state of immunosuppression (16).

We collected the clinical records of the RTPs since their transplantation. Twenty-four RTPs suffered from OC in the first posttransplantation month, 19 of whom had large candidal lesions affecting the oral cavity and pharynx. This follow-up has not been conducted previously and such severe cases of OC were not observed in other studies of RTPs (8-13). This finding may be because of severe immunosuppression in the immediate posttransplantation period, increasing the risk of OC in these patients.

The relationship between the type of immunosuppressant and the prevalence of OC remains controversial. Spolidorio et al. (23) showed that RTPs receiving a CsA-based immunosuppressant regimen have higher salivary levels of Candida species than those treated with FK-506. These findings are quite striking because the immunosuppressive potential of FK-506 is higher than that of CsA. However, we were unable to find any relationship between the prevalence of OC and the different immunosuppressant protocols, similar to the study by Dongari-Bagtzoglou et al. (8).

Immunosuppressants commonly administered to RTPs, such as corticosteroids, adversely affect all aspects of immunity. Moreover, certain antiproliferative medications, such as MMF and azathioprine, can trigger neutropenia, an important predisposing factor for candidiasis (8). In fact, Anees et al. (24) suggested that the use of MMF is associated with a higher rate of clinically apparent OC than the immunosuppressant regimens without this drug. In our study, we observed a significant relationship between the presence of OC and high doses and blood levels of MMF. Although immunosuppressants seemingly elevate the risk of OC, significant associations of their doses and blood levels with higher risk of OC (apart from the MMF dose and blood level) were not demonstrated in this and other studies (8-12). This lack of association could be explained by the need for pharmacologically homogeneous RTP populations to achieve statistically significant results. It would be interesting to analyze the presence of OC in uniform groups of RTPs receiving different immunosuppressant regimens.

Diabetes has been associated with a higher tendency for OC (25,26). However, Dongari-Bagtzoglou et al. (8) did not find any relationship between the presence of diabetes and Candida colonization, colony-forming units, and titers in the oral cavity of recipients of kidney or heart transplants and HCs. Similarly, we found no relationship between the presence of OC and diabetic history. This result may be attributable to the fact that the patients with diabetes in our study had good blood glucose control. Some studies have shown that candidiasis occurs in conditions of poor blood glucose control (25-27). Studies to ascertain the relationships between the blood glucose levels and the prevalence and severity of OC in RTPs are desirable.

Golecka et al. (28) showed that patients with transplants who wear dentures are more likely to suffer from denture stomatitis and angular cheilitis than HCs with dentures. In our study, the clinical forms of OC associated with removable dentures were more frequent in the RTPs than in the HCs. Further, the RTPs who wore dentures were more likely to develop OC. The high prevalence of denture stomatitis in such patients highlights the need for adequate pre transplantation and posttransplantation oral health as well as denture cleaning and adjustment to prevent this infection.

Tobacco is a local risk factor that favors the development of OC. In our study, we observed that tobacco was associated with OC. This may be due to the decrease the amount of saliva produced and the alteration of the antibacterial properties by tobacco habit increasing the colonization of the oral cavity by Candida albicans (29).

In conclusion, we have shown that RTPs have significantly higher prevalence of OC than HCs, although the prevalence is lower than that previously reported. Denture stomatitis is the most frequent form of OC in RTPs, followed by angular cheilitis and pseudomembranous candidiasis. Severe candidiasis is more frequent in the immediate posttransplantation period. The presence of dentures is a major risk factor for OC. Our results emphasize the importance of adequate pretransplantation and posttransplantation oral health and regular denture cleaning and adjustment to prevent this infection in RTPs.
